# Clinical values of diaphragmatic movement in patients with chronic obstructive pulmonary disease

**DOI:** 10.1186/s12890-022-02220-7

**Published:** 2023-01-27

**Authors:** Taehwa Kim, Sungchul Huh, Jae Heun Chung, Yun Seong Kim, Ra Yu Yun, Onyu Park, Seung Eun Lee

**Affiliations:** 1 Division of Respiratory, Allergy and Critical Care Medicine, Department of Internal Medicine, Pusan National University Yangsan Hospital and Pusan National University School of Medicine, Geumo-ro 20, Beomeo-ri, Yangsan-si, Gyeongsangnam-do 50612 Republic of Korea; 2 BioMedical Research Institute for Convergence of Biomedical Science and Technology, Pusan National University Yangsan Hospital, Yangsan, South Korea; 3 Department of Rehabilitation Medicine, Rehabilitation Hospital, Pusan National University Yangsan, Yangsan, South Korea; 4 Pusan National University School of Medicine, Yangsan, South Korea; 5 College of Nursing, Pusan National University, Pusan National University Yangsan Hospital, Yangsan, South Korea

**Keywords:** 6-minute walk test, Diaphragm, Excursion, FEV1, Cut-off value

## Abstract

**Background:**

The limitation of activity due to dyspnea in chronic obstructive pulmonary disease (COPD) patients is affected by diaphragmatic dysfunction and reduced lung function. This study aimed to analyze the association between diaphragm function variables and forced expiratory volume in the first second (FEV1) and to estimate the clinical significance of diaphragm function in the correlation between COPD severity and lung function.

**Methods:**

This prospective, single-center, cross-sectional observational study enrolled 60 COPD patients in a respiratory outpatient clinic. Data for baseline characteristics and the dyspnea scale were collected. Participants underwent a pulmonary function test (PFT), a 6-minute walk test (6MWT), and diaphragm function by ultrasonography.

**Results:**

The right excursion at forced breathing showed the most significant correlation with FEV1 (*r* = 0.370, *p* = 0.004). The cutoff value was 6.7 cm of the right diaphragmatic excursion at forced breathing to identify the FEV1 above 50% group. In the group with a right diaphragmatic excursion at forced breathing < 6.7 cm, modified Medical Research Council (mMRC), St. George's Respiratory Questionnaire and the total distance of 6MWT showed no difference between groups with FEV1 under and above 50% (*p* > 0.05). In the group with ≥ 6.7 cm, mMRC and the total distance of 6MWT showed a significant difference between FEV1 under and above 50% (*p* = 0.014, 456.7 ± 69.7 m vs. 513.9 ± 60.3 m, *p* = 0.018, respectively).

**Conclusion:**

The right diaphragmatic forced excursion was closely related to FEV1, and analysis according to the right diaphragmatic forced excursion-based cut-off value showed a significant difference between both groups. When the diaphragm function was maintained, there was a lot of difference in the 6MWT’s factors according to the FEV1 value. Our data suggest that diaphragmatic function should be performed when interpreting PFT.

**Supplementary Information:**

The online version contains supplementary material available at 10.1186/s12890-022-02220-7.

## Introduction

The most common complaint in respiratory diseases regardless of the disease type is dyspnea [1]. COPD is characterized by worsening dyspnea during movement [2]. COPD restricts various activities of daily living due to shortness of breath, leading to poor quality of life and increased mortality and morbidity [3]. There are many causes of dyspnea; however, for patients with stable COPD, a major contributor is the weakening of the respiratory muscles, excluding conditions such as acute infectious diseases [4].

The diaphragm is the main respiratory muscle, particularly the inspiratory muscles. The weakness of the diaphragm in COPD has been extensively studied. Some studies have reported a significant reduction in diaphragmatic excursion in patients with COPD [5**–**7]. Lung hyperinflation-associated shortening of the diaphragm has traditionally been considered a major cause of diaphragmatic weakness [8]. Also, there were previous studies about diaphragmatic thickness. Diaphragmatic thickness was a factor related to weaning and prognosis in patients under mechanical ventilation [9, 10]. Recently, several studies have reported the clinical value of diaphragm ultrasonography according to COPD severity, and even compared to traditional methods, the diagnostic value of ultrasonography has proven to be reliable and useful [11]. Ultrasonography is also commonly used in medical facilities because it can be carried out anywhere, has no associated radiation risk, and can be used to adequately visualize the structure of the diaphragm [12].

Furthermore, 6MWT is an important tool for assessing exercise capacity and functional status in patients with COPD. Diaphragmatic weakness can impair physical performance, especially the 6MWT [13, 14]. A previous study reported that pulmonary function was significantly correlated with the 6MWT in patients with severe and very severe COPD [15]. The relationship between 6MWT and PFT is a matter of connecting and understanding the respiratory muscles. PFT is used to measure the volume and flow rate of the lungs, and 6MWT is an important test for evaluating the exercise capacity and functional status of patients.

When we summarize the above, PFT correlates with 6MWT in COPD patients [15]. 6MWT can evaluate physical performance of COPD patients. Physical performance can also reflect diaphragmatic weakness [13, 14]. Therefore, PFT correlates with 6MWT, 6MWT reflects physical performance, and physical performance was associated with diaphragmatic weakness. This relationship of PFT and diaphragmatic weakness can be expressed as follows for the patient. If the pulmonary function expressed by PFT is good, or if case which the power and strength of the respiratory muscles are good when PFT remains the same, breathing is more stable. Therefore, understanding the physiological principles of the respiratory muscle performance that establish the relationship these and compensate for this is important for managing the patient’s condition. Through this study, a review of the correlation between the PFT reflecting the 6MWT and diaphragm ultrasound features of respiratory muscle may be helpful to understand the physiological principles of patients with COPD.

Thus, this study aimed to analyze diaphragm movement characteristics using ultrasonography in patients with COPD and clarify its association with pulmonary function.

## Study design and methods

### Study design and participants

This single-center, prospective, cross-sectional observational study recruited participants from a tertiary hospital outpatient respiratory clinic between April 2020 and April 2021. The inclusion criteria were: 1) patients 18 years old or older diagnosed with COPD by a pulmonologist; COPD diagnostic criterion was a post-bronchodilator FEV1/forced vital capacity (FVC) ratio < 0.70 based on the Global Initiative for Chronic Obstructive Lung Disease (GOLD), 2) patients who could maintain the required posture for diaphragm function measurement by ultrasonography and stable breathing during the examination such as 6MWT. Patients unable to cooperate with the examination and unstable patients requiring immediate medical intervention were excluded. Patients with interstitial lung disease featured on chest computed tomography (CT) that could affect diaphragm movement were also excluded.

Sixty-nine patients were enrolled, six of whom with combined interstitial lung disease on CT were excluded. Two patients were lost to follow-up, and one died before all examinations were completed. Finally, 60 patients completed all examinations for the study protocol and were included in the analysis.

All patients provided informed consent before participating in the study. Each patient’s clinical information was collected from four domains: pulmonary function, exercise capacity, body composition, and diaphragm function. Pulmonary function was evaluated through spirometry, MIP, and maximal expiratory pressure (MEP). Exercise capacity and body composition were assessed using the 6MWT and bioelectrical impedance analysis (BIA). Diaphragm dysfunction is defined as loss of muscle contractility [16]. To evaluated diaphragm dysfunction, we was assessed using ultrasonography in both the M-mode and B-mode for excursion and thickness, respectively.

### Assessments

For patients who had performed a PFT within 1 month of participating in the study, the previous results were used and no retesting was performed. Patients who had no available PFT results within 1 month of participating in this study were reevaluated after enrollment. The Carefusion Vmax 20 (VIASYS Healthcare Inc. Sensormedics; Yorba Linda, CA, USA) was used for PFTs and FEV1, FVC, diffusing capacity of the lungs for CO, and total lung capacity were measured using the body plethysmography test. Regarding spirometry, the patients sat in a small booth and breathed into a mouthpiece. One technical expert from the Department of Respiratory Medicine conducted all the tests to maintain the consistency of the results.

MIP (PONY FX, COSMED Inc.; Rome, Italy) and MEP (PONY FX, COSMED Inc.; Rome, Italy) were measured in the sitting position using a portable mouth pressure meter. Three consecutive MIP and MEP measurements were taken, and the best result was recorded. The PFT was measured in a sitting position. A flanged mouthpiece was applied to the short and rigid tube of the measuring instrument and air leakage was checked around the mouthpiece before testing. The test was performed by an experienced examiner who has conducted the test for more than 8 years. MIP was measured by exhaling as deep as possible and inhaling as hard as possible for at least 1.5 s. MEP was measured by inhaling as deep as possible and exhaling as hard as possible for at least 1.5 s. Both measurements were made three times, and patients recovered to normal breathing patterns with at least a minute of break between measurements. The highest of the three measurements was recorded [17].

The 6MWT was performed according to the American Thoracic Society standards under the direction of a well-trained respiratory therapist at a 30 m indoor walking course [18]. Patients were encouraged by the instructor every minute and were allowed to rest or quit the test at any point. We measured the total distance and peripheral saturation with the portable oxygen meter. The patients’ body compositions were estimated indirectly using the BIA from a supine position (InBody S10, InBody, Co. Ltd., Seoul, Korea).

Diaphragm function was assessed using ultrasonography (LOGIQ E9, GE Healthcare; Chicago, IL, USA) obtained from both supine and sitting positions. It is generally accepted that there are positional differences in diaphragm contractility. The effects of gravitational loading on the diaphragm length-tension and body position-mediated changes in intra-abdominal pressure may explain the differences found. Not only that there is also a difference in the excursion between right and left. The excursion of the right diaphragm shows a lower value than that of the left diaphragm because the liver in the abdominal cavity restricts the movement of the right diaphragm. We also measured the diaphragm function in two positions based on this information. The supine position involved lying on the back or with the face upward while the sitting position was semi-seated (45–60 degrees). Both M-mode and B-mode imaging were used to evaluate diaphragmatic excursion and thickness, respectively. The mid-clavicular line and the liver were used as anatomical landmarks on the right side and the spleen on the left side to visualize the diaphragm in the M-mode. B-mode ultrasonography was used to measure the diaphragmatic thickness at the bilateral zone of apposition [19]. The diaphragm thickness was measured during quiet spontaneous breathing without peak inspiratory or expiratory maneuvers. The diaphragmatic thickness fraction was calculated as the difference between thickness at the end of inspiration and thickness at the end of expiration divided by thickness at the end of expiration x 100. The diaphragmatic excursion was measured as follows. The highest position of the diaphragm movement taken by the M-mode was considered to be the end-expiratory phase, whereas the lowest position was considered as the end-inspiratory phase.

The dyspnea scale used St. George's Respiratory Questionnaire (SGRQ) and the modified Medical Research Council scale (mMRC scale). The SGRQ is a self-administered questionnaire with 76 items [20]. This can identify the patient’s symptoms and the activities of daily life. mMRC scale is most commonly used in the assessment of dyspnea in chronic respiratory diseases and is a very useful and unrecognized dyspnea scale [21].

### Statistical analysis

The data were analyzed using IBM SPSS (version 27.0; Chicago, IL, USA). The level of significance was set at *p* < 0.05. Descriptive statistics, including numbers, percentages, means, and standard deviations, were used to summarize each variable (demographics, PFTs, 6MWT, and diaphragmatic ultrasound results). The results were analyzed by independent t-test, cross-analysis, and frequency analysis. The correlation between the variables was analyzed by Pearson’s Correlation Coefficient, which confirmed the linear relationship between two variables using a scatterplot. The cut-off value was calculated using the receiver operating characteristic (ROC) curve analysis. The reference plane was 0.5 or more in the ROC curve, and the *p*-value < 0.05; hence, this result was adopted. Consequently, the cut-off value was confirmed when sensitivity and specificity were plotted in a line chart, which is the point where the two graphs meet.

### Ethics statement

We certify that all applicable institutional and governmental regulations concerning the ethical use of human volunteers were followed throughout this study. The study procedures were reviewed and approved by our Pusan National University Yangsan Hospital Institutional Review Board [IRB No. 05–2020-217].

## Results

### FEV1 and diaphragm function

**Fig. 1:**
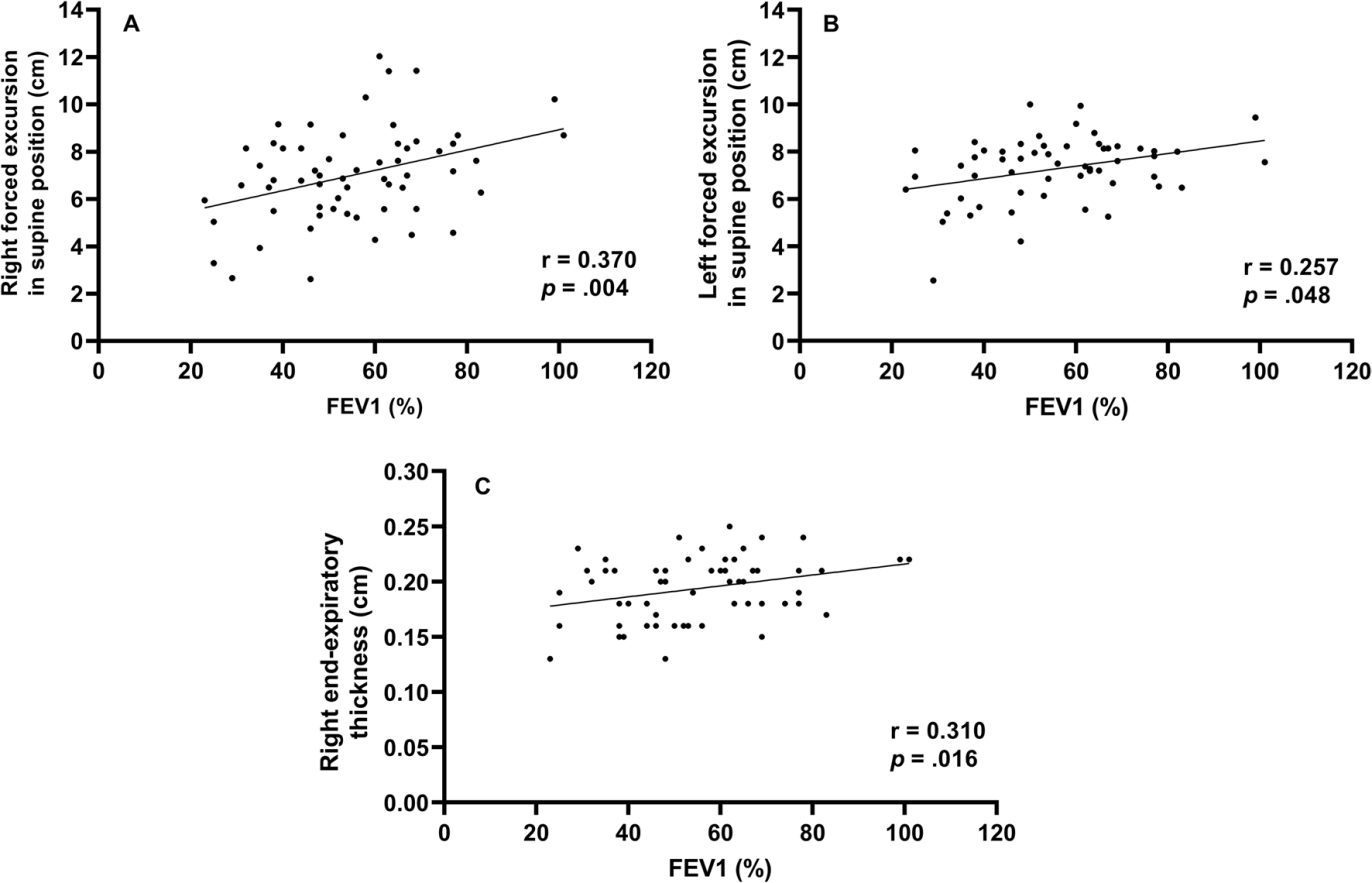
Correlation between forced expiratory volume in 1 s and diaphragm function Right forced excursion, and left forced excursion in the supine position and right end-expiratory thickness were correlated to forced expiratory volume in 1 s

We assessed whether diaphragm function was associated with FEV1 (Fig. 1). In the total group analysis, both diaphragmatic excursion and thickness were associated with FEV1. However, the diaphragmatic excursion was more associated with FEV1 than thickness. Diaphragmatic excursion during forced breathing and in the supine position had a greater association with FEV1 than breathing at rest and in the sitting position. Additionally, when comparing the right and left under the same conditions, the right was more significant during forced breathing and in the supine position (*r =* 0.370, *p* = 0.004,). Moreover, diaphragmatic thickness at right end-expiration was associated with FEV1. In summary, right (*r =* 0.370, *p* = 0.004) and left (*r =* 0.257, *p* = 0.048) diaphragmatic excursion during forced breathing in the supine position and diaphragmatic thickness at right end-expiration (*r =* 0.310, *p* = 0.016) were significantly associated with FEV1.

### Diaphragmatic function and BMI (body mass index)

To evaluate the function of the diaphragm muscle [22], the diaphragmatic excursion was measured at rest and during forced expiration (Supplement Table 1). In 60 patients, diaphragmatic excursion at rest in the supine position was 3.5 cm ± 1.2 on the right side and 3.5 cm ± 1.2 on the left side. During forced breathing, diaphragmatic excursion in the supine position was 6.9 cm ± 2.0 on the right side and 7.6 cm ± 1.6 on the left side. The total percent body fat was 24.2% ± 6.9. Segmental lean mass analysis was performed by direct segmental multi-frequency BIA. The lean mass was 90.5% ± 9.7 on the right arm, 88.1% ± 9.2 on the left arm, 94.5% ± 5.8 on the trunk, 95.7% ± 131.3 on the right leg, and 9.51% ± 8.8 on the left leg.

### Cutoff value-associated characteristics

The ROC curve analysis of the diaphragm function variables was performed to identify the cutoff value for differentiating between FEV1 ≥ 50% and < groups. The cutoff value was ≤ 6.7 cm on the right diaphragmatic excursion at forced breathing with an area under the curve of 0.5 or more and *p*-value was 0.043. Right diaphragmatic excursion during forced breathing was less than the cut-off value of 6.7 cm for 26 patients and ≥ 6.7 cm for 43 patients (Table 1). There were no differences in age, sex, or smoking history between the two groups. The dyspnea scales such as mMRC, SGRQ, and GOLD were not significantly different between both groups. There were no differences in body mass index, percent body fat, or lean mass of the right or left legs between the groups. However, among the pulmonary function indicators, there were significant differences between the two groups. Specifically, FEV1, FVC, and MIP were significantly different (< 6.7 cm group vs. ≥ 6.7 cm group, FEV1: 49.2% ± 16.2 vs. 59.5% ± 17.2, *p* = 0.021; FVC: 76.2% ± 19.1 vs. 86.0% ± 15.5, *p* = 0.032; MIP: 67.4 cm H_2_O ± 25.1 vs. 86.5 cm H_2_O ± 28.7, *p* = 0.010). Concerning the 6MWT, there was a significant difference in SpO2 before 6MWT and the number of interruptions (SpO2 before 6MWT: 94.1% ± 2.7 vs. 95.3% ± 1.6, *p* = 0.038; number of interruptions: 4 [15.4%] vs. 0 [0%], *p* = 0.018). The left diaphragmatic excursion during forced breathing was also different between the two groups (6.8 cm ± 1.5 vs. 7.6 cm ± 1.3, *p* = 0.022) as well as the diaphragmatic thickness during right end-inspiration (0.3 cm ± 0.1 vs. 0.4 cm ± 0.1, *p* = 0.006). In addition, the ROC ≥ 6.7 cm group left diaphragmatic excursion was also measured with a value greater than that of the ROC < 6.7 cm group.

**Table 1:** Patient characteristics according to the cutoff value of right diaphragm forced excursion

	Total ***N=***60	< 6.7 cm ***n=***26	≥ 6.7 cm ***n=***34	***p***-value
Age (years)	69.7 ± 7.2	68.5 ± 8.0	70.5 ± 6.5	0.298
Sex (male)	59 (98.3)	26 (100)	33 (97.1)	0.378
Smoking				0.925
Never	26 (43.3)	12 (46.2)	14 (41.2)	
Current	10 (16.7)	4 (15.4)	6 (17.6)	
Former	24 (40.0)	10 (38.5)	14 (41.2)	
mMRC				0.351
0	0	0	0	
1	21 (43.3)	7 (26.9)	14 (41.2)	
2	31 (51.7)	14 (53.8)	17 (50.0)	
3	8 (13.3)	5 (19.2)	3 (8.8)	
SGRQ	33.1 ± 15.2	35.5 ± 15.0	31.2 ± 15.3	0.286
GOLD				0.085
1 (mild)	5 (8.3)	1 (3.8)	4 (11.8)	
2 (moderate)	31 (51.7)	12 (46.2)	19 (55.9)	
3 (severe)	20 (33.3)	9 (34.6)	11 (32.4)	
4 (very severe)	4(6.7)	4 (15.4)	0 (0)	
BMI (kg/m^2^)	23.0 ± 3.1	22.8 ± 3.0	23.1 ± 3.2	0.681
Percent body fat (%)	24.2 ± 6.9	24.4±7.5	25.7±6.5	0.463
PFT				
FEV1 (%)	55.1 ± 17.4	49.2 ± 16.2	59.5±17.2	**0.021**
FVC (%)	81.7 ± 17.7	76.2 ± 19.1	86.0 ± 15.5	**0.032**
FEV1/FVC	48.2 ± 14.5	47.2 ± 15.2	49.0 ± 14.1	0.629
Maximum inspiratory pressure (cmH_2_O)	78.4 ± 28.6	67.4 ± 25.1	86.5 ± 28.7	**0.010**
Maximum expiratory pressure (cmH_2_O)	83.1 ± 27.5	83.2 ± 32.0	83.0 ± 24.2	0.977
Peak expiratory flow rate (L/min)	245.2 ± 119.7	227.7 ± 106.8	258.9 ± 129.0	0.335
6MWT				
Total distance of 6MWT (m)	461.1 ± 121.3	416.8 ± 159.9	493.7 ± 68.6	**0.031**
SpO2 before 6MWT (%)	94.8 ± 2.2	94.1 ± 2.7	95.3 ± 1.6	**0.038**
SpO2 after 6MWT (%)	88.6 ± 5.6	88.4 ± 5.7	88.8 ± 5.5	0.784
Interruption of the 6MWT (n)		4 (15.4)	0 (0)	**0.018**
Diaphragm (cm)				
Right forced excursion*	7.0 ± 1.2	5.3 ± 1.2	8.3 ± 1.4	**<0.001**
Left forced excursion*	7.3 ± 1.4	6.8 ± 1.5	7.6 ± 1.3	**0.022**
Right thickness, end-expiration	0.2 ± 0.0	0.2 ± 0.0	0.2 ± 0.0	0.256
Right thickness, end-inspiration	0.3 ±0.1	0.3 ± 0.1	0.4 ± 0.1	**0.006**
Right thickness Fraction (%)	76.8 ± 31.2	66.0 ± 30.4	85.1 ± 29.7	**0.017**
Left thickness Fraction (%)	85.9 ± 157.7	66.8 ± 27.0	100.5 ± 208.3	0.417

Abbreviations: *6MWT* 6-minute walk test, *BMI* Body mass index, *FEV1* Forced expiratory volume in the first second, *FVC* Forced vital capacity, *GOLD* Global Initiative for Chronic Obstructive Lung Disease, *mMRC* Modified Medical Research Council, *SGRQ* St. George's Respiratory Questionnaire, *PFT* Pulmonary function test; *supine position

### Subgroup characteristics according to FEV1

To identify the clinical significance of diaphragm function with the relationship between lung function, and COPD severity, the two groups classified as a right diaphragmatic excursion at 6.7 cm of forced breathing were further divided into groups based on FEV1 (< 50% or ≥ 50%) (Table 2). There were significant differences in age (65.0 ± 7.8 years vs. 72.7 ± 6.2 years, *p* = 0.011), the GOLD score (*p* < 0.001), FEV1/FVC (40.1% ± 14.7 vs. 55.%4 ± 11.4, *p* = 0.007), peak expiratory flow rate (183.3 L/min ± 80.4 vs. 275.8 L/min ± 113.8, *p* = 0.027), SpO2 after the 6MWT (85.9% ± 6.5 vs. 91.5% ± 2.2, *p* = 0.011), and left diaphragmatic excursion during forced breathing (6.2 cm ± 1.6 vs. 7.4 cm ± 1.0, *p* = 0.038).

**Table 2:** Subgroup characteristics according to FEV1 50%

	< 6.7 cm ***n=***26			≥ 6.7 cm ***n=***34		
	FEV1 < 50% ***n=***14	FEV1 ≥ 50% ***n=***12	***p***-value	FEV1 < 50% ***n=***12	FEV1 ≥ 50% ***n=***22	***p***-value
Age (years)	65.0 ± 7.8	72.7 ± 6.2	**0.011**	69.8 ± 7.2	70.9 ± 6.2	0.627
Sex (male)	14 (100)	12 (100)		11 (91.7)	22 (100)	0.169
Smoking			0.292			0.655
Never	6 (42.9)	6 (50.0)		4 (33.3)	10 (45.5)	
Current	1 (7.1)	3 (25.0)		3 (25.0)	3 (13.6)	
Former	7 (50.0)	3 (25.0)		5 (41.7)	9 (40.9)	
mMRC			0.407			**0.014**
0	0	0		0 (0)	0	
1	3 (21.4)	4 (33.3)		1 (8.3)	13 (59.1)	
2	7 (50.0)	7 (58.3)		9 (75.0)	8 (36.4)	
3	4 (28.6)	1 (8.3)		2 (16.7)	1 (4.5)	
SGRQ	38.3 ± 13.8	32.2 ± 16.2	0.306	37.5 ± 14.4	27.8 ± 15.1	**0.080**
GOLD			**<0.001**			**<0.001**
1 (mild)	0 (0)	1 (8.3)		0 (0)	4 (18.2)	
2 (moderate)	1 (7.1)	11 (91.7)		1 (8.3)	18 (81.8)	
3 (severe)	9 (64.3)	0 (0)		11 (91.7)	0 (0)	
4 (very severe)	4 (28.6)	0 (0)		0 (0)	0 (0)	
BMI (kg/m^2^)	22.2 ± 3.4	23.5 ± 2.3	0.302	22.9 ± 2.6	23.3 ± 3.5	0.753
Percent body fat (%)	22.7±8.1	26.2±6.5	0.245	26.2±7.5	25.4±6.1	0.764
PFT						
FEV1	37.0 ± 8.7	63.4 ± 9.9	**<0.001**	41.8 ± 5.6	69.2 ± 13.0	**<0.001**
FVC	73.5 ± 21.4	79.3 ± 16.3	0.454	86.2 ± 14.2	85.9 ± 16.5	0.958
FEV1/FVC	40.1 ± 14.7	55.4 ± 11.4	**0.007**	34.7 ± 5.5	56.8 ± 10.813	**<0.001**
Maximum inspiratory pressure (cmH2O)	70.2 ± 29.4	64.4 ± 20.5	0.572	72.5 ± 32.1	94.1 ± 24.1	**0.034**
Maximum expiratory pressure (cmH2O)	80.9 ± 28.4	85.7 ± 36.7	0.718	76.0 ± 27.2	86.8 ± 22.1	0.218
Peak expiratory flow rate (L/min)	183.3 ± 80.4	275.8 ± 113.8	**0.027**	166.7 ± 61.5	307.1 ± 129.5	**0.002**
6MWT						
Total distance of 6MWT (m)	404.3 ± 164.0	432.7 ± 160.8	0.668	456.7 ± 69.7	513.9 ± 60.3	**0.018**
SpO2 before 6MWT (%)	93.3 ± 2.9	95.3 ± 1.7	0.066	94.4 ± 1.7	95.8 ± 1.4	**0.014**
SpO2 after 6MWT (%)	85.9 ± 6.5	91.5 ± 2.2	**0.011**	85.7 ± 5.1	90.5 ± 5.0	**0.013**
Interruption of the 6MWT (n)	2 (14.3)	2 (16.7)	0.867	0 (0)	0 (0)	
Diaphragm (cm)						
Right forced excursion*	5.0 ± 1.4	5.6 ± 0.8	0.188	7.8 ± 0.8	8.6 ± 1.5	0.108
Left forced excursion*	6.2 ± 1.6	7.4 ± 1.0	**0.038**	7.7 ± 1.3	7.6 ± 1.3	0.812
Right thickness, end-expiration	0.2182 ± 0.0	0.2 ± 0.0	0.192	0.2 ± 0.0	0.2 ± 0.0	**0.020**
Left thickness, end-inspiration	0.1 ± 0.1	0.3 ± 0.1	0.920	0.3 ± 0.1	0.4 ± 0.1	0.284
Rigth thicknsee Fraction (%)	72.1 ± 38.6	58.9 ± 15.3	0.254	89.4 ± 31.8	82.8 ± 28.9	0.542
Left thickness Fraction (%)	71.4 ± 32.2	61.5 ± 19.3	0.358	67.3 ± 27.8	118.7 ± 258.5	0.500

Abbreviations: *6MWT* 6-minute walk test, *BMI* Body mass index, *FEV1* Forced expiratory volume in the first second, *FVC* Forced vital capacity, *GOLD* Global Initiative for Chronic Obstructive Lung Disease, *mMRC* Modified Medical Research Council, *SGRQ* St. George's Respiratory Questionnaire, *PFT* Pulmonary function test; *supine position

After dividing the total patients based on ROC < 6.7, each group was divided into FEV1 50% standard and compared the characteristics of each subgroup.

When the group with the right diaphragmatic excursion ≥ 6.7 cm was further divided into subgroups according to FEV1 (< 50% or ≥ 50%) and analyzed, mMRC, GOLD score, FEV1/FVC, MIP, peak expiratory flow rate, 6MWT, SpO2 before and after the 6MWT, and right diaphragmatic thickness at end-expiration subgroups were significantly different between the two groups.

## Discussion

This study contains the following: 1) evidence that FEV1 is significantly correlated with diaphragm movement, 2) cutoff values for diaphragm movement in patients with COPD, and 3) evidence to support the claim that the function of the diaphragm should be considered when interpreting the patient’s condition based on their FEV1.

First, FEV1 was significantly correlated with diaphragm movement. Studies on the relationship between the diaphragm and pulmonary function in patients with COPD are ongoing and have consistently reported that the severity of COPD and diaphragm function are closely related. Some previous studies have evaluated the direct relationship between FEV1 and diaphragm function [23, 24].

The results of this study is also consistent with those of previous studies showing that diaphragm movement and FEV1 are related. However, beyond the findings of previous results [23], in our study, diaphragmatic excursion and thickness were found to be more correlated to FEV1 on the right side than on the left side.

Like the previous study that the thickness of the diaphragm is related to the ventilator weaning mechanical ventilation [9, 10], this result has confirmed that the right diaphragm thickness was significantly related not only to the weaning of the ventilator and the prognosis of the patient but also to FEV1.

Second, we provided a cutoff value for a right diaphragmatic forced excursion in patients with COPD. Although there are studies that have presented a reference [23] value for healthy persons, the significant contribution of this study is the proposed reference value for patients with COPD.

We analyzed the correlation using Pearson’s correlation coefficient and confirmed the factors of diaphragmatic function-related components side (right, left), thickness, and excursion that were most-related to FEV1. Among them, Rt. forced excursion (supine), Lt. forced excursion (supine) and Rt. end-expiratory thickness showed meaningful *p*-value in association with FEV1. In addition, these three factors were analyzed in the linear relationship with the scattered plot and showed a proportional relationship between FEV1. Finally, when all factors related to the diaphragmatic function were analyzed, the right forced excursion was statistically determined as the most meaningful factor in relation to FEV1. We also obtained the cut-off value of 6.7 cm through the ROC curve.

The range in diaphragmatic excursion values varies considerably depending on the patient’s condition. A previous study has suggested normal values based on sex and the side of the diaphragm using healthy individuals. When breathing deeply, the right diaphragmatic excursion was 7 cm ± 1.1 in men and 5.7 cm ± 1 in women (*p* < 0.001) and the left diaphragmatic excursion were 7.5 cm ± 0.9 and 6.4 cm ± 1 in men and women, respectively (*p* < 0.01) [23]. In our study, we also assessed excursion during deep breathing to provide a cut-off value for patients with COPD.

When analyzed by dividing them into two groups based on a cut-off value, the following evaluation factors showed significant differences (*p* < 0.05): FEV1, FVC, MIP, left forced excursion, right diaphragmatic thickness during end-inspiration, 6MWT, the SpO2 before and after 6MWT, and interruption of the 6MWT.

These factors can be broadly divided into PFT-related and performance-related factors. As mentioned above, PFT-related factors such as MIP, left diaphragmatic forced excursion and right diaphragmatic thickness during end-inspiration were lower in the < 6.7 cm group. Moreover, the SpO2 level before the 6MWT was lower in the < 6.7 cm group, the overall 6MWT was shorter, and there were many interruptions in the 6MWT. These factors might reflect activity as a performance evaluation factor. Although generalizability is limited given the few patients and the fact that all the participants were outpatients who could walk; these results may reflect an actual patient’s status. However, these findings are intended for patients who can walk, suggesting that the cut-off value of 6.7 cm may be reliable in this population.

Finally, results concerning the degree of pulmonary function and correlations with the diaphragmatic movement were noteworthy. The two groups were analyzed based on the right diaphragmatic forced excursion (6.7 cm) and divided into subgroups based on FEV1 (< 50% vs. ≥ 50%). As a result, in the group that had maintained diaphragm function (≥ 6.7 cm), the MIP, portable peak flow meter, 6MWT, SpO2 before and after the 6MWT, and right diaphragmatic thickness at end-expiration were different between the two FEV1 groups. In summary, the difference between the two FEV1 groups was large when diaphragm function was maintained; when it was not maintained, there were no differences between the two FEV1 groups. Therefore, even in patients who maintained their FEV1 > 50%, when diaphragm function deteriorated, the patient’s 6MWT, SpO2 before and after the 6MWT were less predictable (they either deteriorated or were maintained). The patients whose FEV1 decreased < 50%, if the diaphragm function was maintained, the 6MWT could be better than that in patients with an FEV1 ≥ 50% and a reduced diaphragm function.

In conclusion, when interpreting a patient’s condition based on FEV1, it is important to assess diaphragm function, since the effect of the FEV1 value on the patient depends on how well the diaphragm function has been maintained.

## Conclusion

In this study, when the diaphragm function was maintained, there were significant differences in MIP, peak expiratory flow rate, 6MWT, SpO2 before and after the 6MWT, and right diaphragmatic thickness at end-expiration according to FEV1 in patients with COPD. Even if the diaphragm function was not maintained, because there are still differences in the FEV1, it may be beneficial to consider diaphragmatic function measured by right diaphragm excursion as an additional indicator of function beyond the FEV1. Therefore, it can be clinically helpful to check whether the diaphragm is functioning properly when determining a patient’s condition based on FEV1.

## Supplementary Information

**Additional file 1.**

## Data Availability

The datasets used and/or analysed during the current study are available from the corresponding author on reasonable request.

## Abbreviations

COPD: Chronic obstructive pulmonary disease
PFT: Pulmonary function test
6MWT: 6-minute walk test
FEV1: Forced expiratory volume in the first second
MIP: Maximal inspiratory pressure
ICD-11: International Classification of Diseases 11TH
FVC: Forced vital capacity
GOLD: Global Initiative for Chronic Obstructive Lung Disease
CT: Computed tomography
MEP: Maximal expiratory pressure
BIA: Bioelectrical impedance analysis
mMRC: Modified Medical Research Council
ROC: Receiver operating characteristic
BMI: Body mass index
SGRQ: St. George's Respiratory Questionnaire
