# Supplementary material for: Clinical values of diaphragmatic movement in patients with chronic obstructive pulmonary disease
Source: BMC Pulm Med. 2023 Jan 27;23:33. doi: 10.1186/s12890-022-02220-7 (PMC9881315; doi:10.1186/s12890-022-02220-7)
Supplement: Supplementary file 1 — Additional file 1. [file 12890_2022_2220_MOESM1_ESM.docx]

Supplement Table 1. Diaphragm Function (total patients)

| **Variable** | **Total**  **N=60** |
| --- | --- |
| *Diaphragm*  *Supine position*  Rt. diaphragmatic excursion (forced breathing)  Lt. diaphragmatic excursion (forced breathing)  Rt. diaphragmatic excursion (rest breathing)  Lt. diaphragmatic excursion (rest breathing)  Rt. diaphragmatic thickness, end-expiration  Rt. diaphragmatic thickness, end-inspiration  Rt. thickness Fraction  Lt. diaphragmatic thickness, end-expiration  Lt. diaphragmatic thickness, end-inspiration  Lt. thickness Fraction | 7.0 ± 1.2  7.3 ± 1.4  3.5 ± 1.2  3.5 ± 1.2  0.2 ± 0.0  0.3 ± 0.1  76.8 ± 31.2  0.2 ± 0.0  0.3 ± 0.1  66.2 ± 27.9 |
| *Sitting position*  Rt. diaphragmatic excursion (forced breathing)  Lt. diaphragmatic excursion (forced breathing)  Rt. diaphragmatic excursion (rest breathing)  Lt. diaphragmatic excursion (rest breathing) | 8.7 ± 2.4  8.1 ± 1.4  4.0 ± 1.2  4.0 ± 1.5 |

Lt, left; Rt, right
